# Stability of Turoctocog Alfa, a Recombinant Factor VIII Product, during Continuous Infusion In Vitro

**DOI:** 10.1055/s-0040-1719082

**Published:** 2020-11-06

**Authors:** Masahiro Takeyama, Anne Mette Nøhr, Debra Pollard

**Affiliations:** 1 Department of Pediatrics, Nara Medical University, Kashihara, Japan; 2 Novo Nordisk A/S, Biopharm Project Offices, Gentofte, Denmark; 3 Katharine Dormandy Haemophilia and Thrombosis Centre, Royal Free London NHS Foundation Trust, London, United Kingdom

**Keywords:** continuous infusion, factor VIII, hemophilia A, potency, stability, turoctocog alfa

## Abstract

**Objective** Turoctocog alfa is a recombinant factor VIII (rFVIII) for the prevention and treatment of bleeding in patients with hemophilia A, including those undergoing surgery and invasive medical procedures. This in vitro study evaluated the physical and chemical stability of turoctocog alfa during continuous infusion (CI) over 24 hours at 30°C.

**Materials and Methods** The study was performed at 30°C ( ± 2°C). A CI system with pump speed set at either 0.6 or 1.5 mL/h was used to evaluate the stability of three turoctocog alfa strengths (500, 1,000, and 3,000 IU), equating to doses of 1.1 to 16.1 IU/h per kilogram of body weight. The following parameters were evaluated at selected time points between 0 and 24 hours: appearance of solution, clarity, pH, potency, purity, content, total high molecular weight proteins (HMWPs), and oxidized rFVIII.

**Results** The mean potency of turoctocog alfa was maintained within the predefined acceptance criteria during CI for both pump speeds with all three strengths at 6, 12, or 24 hours (500 IU: ≥484 IU/vial; 1,000 IU: ≥1,014 IU/vial; and 3,000 IU: ≥3,029 IU/vial). Furthermore, the appearance of solution, clarity, pH, purity, content of turoctocog alfa, total HMWP, and oxidized forms were also within the predefined limits, and comparable to the reference samples (time = 0 hours) for the pump speeds and product strengths assessed.

**Conclusion** Physical and chemical stability of turoctocog alfa was maintained during CI over 24 hours. There was only minor degradation or changes in any of the parameters tested. Potency was within the prespecified acceptance limits throughout 24 hours of infusion. These findings confirm the suitability of turoctocog alfa for CI.

## Introduction

Patients with hemophilia A may require major surgery, including elective orthopedic interventions due to chronic hemophilic arthropathy that develops following recurrent joint bleeds. 1 2 To provide hemostatic control and protect against an increased risk of blood loss during major surgery, patients require peri- and postoperative correction of their low or missing factor VIII (FVIII) level by administration of a FVIII product. The World Federation of Hemophilia (WFH) guidelines suggest that FVIII concentrates should be infused by slow intravenous bolus injection at a maximum rate of 3 mL/min in adults and 100 IU/min in young children, or according to the manufacturer's guidelines. 3

Continuous infusion (CI) is an alternative strategy that avoids FVIII concentration peaks and suboptimal troughs. 3 The safety and efficacy of CI of FVIII for major surgery has been shown in a variety of studies, including for joint arthroplasties and synovectomies. 4 5 6 7 8 9 Benefits of CI over bolus injection of FVIII products, such as constant factor levels and reduced FVIII consumption, have also been demonstrated in patients with hemophilia in surgical settings. For example, a prospective controlled trial demonstrated less dangerous drops in FVIII levels and a 36% reduction in total factor use for 13 days of treatment with CI, compared with bolus FVIII infusions. 4

A key requirement of FVIII concentrates for CI is that they are stable under the conditions of administration and over the duration of administration. Stability of various FVIII products have been evaluated under a range of storage conditions, including those usually encountered during CI. 10 11 12 Continuous infusion administration techniques do, however, vary. 13 14 In the hospital setting, infusion pump speeds of 0.4 to 1.5 mL/h have been used to achieve doses of 1 to 10 IU/h/ kg of body weight. 13 14 15

Turoctocog alfa (NovoEight, Novo Nordisk A/S, Bagsværd, Denmark) is a recombinant FVIII (rFVIII) product with a truncated B domain, for the prophylaxis and treatment of bleeds, and for use during surgery in patients with hemophilia A; it is available in six strengths ranging from 250 to 3,000 IU/vial. 16 17 18 19 20 Turoctocog alfa has been shown to be stable when stored at high temperature and high humidity. 11 21 In addition, under conditions similar to those encountered during CI, reconstituted samples of turoctocog alfa were stable and retained >83% of activity after 24 hours at 30°C. 21 The aim of this in vitro study was to evaluate the physical and chemical stability of reconstituted turoctocog alfa at a range of concentrations, and at high- and low-pump speeds over 24 hours CI at 30°C.

## Materials and Methods

### Continuous Infusion Test System

The CI test system comprised a BD Medical Plastipak 50-mL sterile polypropylene Luer-Lok syringe (Becton Dickinson & Company Ltd., Drogheda, Ireland) attached to a 200-cm standard polyethylene Original-Perfusor line Luer-Lok (B Braun, Melsungen AG, Melsungen, Germany), connected to a Perfusor Space automatic infusion pump (B Braun). There was no pretreatment of syringes or infusion tubes. Samples were collected in Immuno Tubes MiniSorp (Thermo Fisher Scientific, Waltham, Massachusetts, United States) and immediately stored at –80°C until analysis.

### Sample Preparation and Analytical Setup

The following two experiments were conducted: (1) one each at a pump speed of 1.5 and 0.6 mL/h. For each experiment, the required number of turoctocog alfa vials were reconstituted separately and pooled. The reconstituted drug product was not protected from direct light. Turoctocog alfa was reconstituted to 4.3 mL using a 5-mL syringe with 0.9% sodium chloride, according to the manufacturer's recommendations. 17 Since the manufacturer recommends using reconstituted turoctocog alfa within 24 hours if stored at 2 to 8°C or 4 hours if stored at ≤30°C, 17 stability was assessed over 24 hours of CI at 30°C. For each experiment, three vial strengths of turoctocog alfa were evaluated (500, 1,000, and 3,000 IU, equating to concentrations of 125, 250, and 750 IU/mL) using identical pumps and test systems. For the pump speeds assessed, this equated to turoctocog alfa doses of 1.1 to 16.1 IU/h/kg body weight (using a standardized body weight of 70 kg; Table 1 ).

**Table 1:** Turoctocog alfa dosages evaluated in study

Turoctocog alfa vial strength (strength postreconstitution)	Calculation a	Study dosage (IU/h/kg of BW)
500 IU (125 IU/mL)	125 IU/mL × 0.6 mL/h/BW	1.1
	125 IU/mL × 1.5 mL/h/BW	2.7
1,000 IU (250 IU/mL)	250 IU/mL × 0.6 mL/h/BW	2.1
	250 IU/mL × 1.5 mL/h/BW	5.4
3,000 IU (750 IU/mL)	750 IU/mL × 0.6 mL/h/BW	6.4
	750 IU/mL × 1.5 mL/h/BW	16.1

Abbreviation: BW, body weight.

a BW estimated at 70 kg.

For both pump speeds assessed, samples during CI were taken for up to 24 hours. Under aseptic conditions in both experiments, sterile syringes were filled with reconstituted turoctocog alfa that connected to an Original-Perfusor Line and then mounted in the infusion pump. At time point 0 hours, a manual bolus sample was taken immediately after the syringe/tube was filled. During CI, samples were collected at 6 hours (range: 0–6 hours), 12 hours (range: 6–12 hours), and 24 hours (range: 12–24 hours). The experiments at different pump speeds were conducted on different days. The study was performed at 30°C ( ±  2°C), and samples were stored at −80°C until analysis.

### Analyses

The study was performed according to International Council for Harmonisation guidelines. 22 The parameters reported were those from the drug product specification, as these are known to be parameters that indicate stability and are susceptible to change during CI at 30°C and/or those likely to influence product quality. Unless stated otherwise, the samples at each time point were pooled and evaluated against a reference sample (time = 0 hour). The results from the analyses below were calculated using appropriate statistical methods 22 ; potency was analyzed using PLA 3.0 software (Stegmann Systems GmbH, Rodgau, Germany) and Microsoft Excel (Microsoft, Redmond, Washington, United States); results were visualized and validated using JMP Statistical Software (SAS Institute Inc., Cary, North Carolina, United States).

### Appearance/Clarity of Solution

The syringe, infusion tube, and turoctocog alfa solution were visually evaluated at 0 and 24 hours. 23 The appearance was considered unchanged at 24 hours if the syringe, infusion tube, and reconstituted turoctocog alfa drug product did not show changes in transparency, color, sedimentation, presence of foreign insoluble matter, or other changes, when compared with the visual description at 0 hours.

### pH

The pH of the samples was assessed at 0 and 24 hours according to the following country-specific standards: European Pharmacopoeia (section 2.2.3), U.S. Pharmacopeia (section 791), and Japanese Pharmacopeia (section 2.54) pH determination. 24 25 26

### Potency

Potency was investigated using the chromogenic kit Coamatic FVIII (Chromogenix, Instrumentation Laboratory, Bedford, Massachusetts, United States) on the ACL Elite Pro analyzer (Instrumentation Laboratory), in accordance with European Pharmacopoeia Assay of human coagulation FVIII using a product-specific standard as calibrator. 27 A product-specific standard (reconstituted in 0.9% sodium chloride) and turoctocog alfa samples from the test systems underwent the following three dilution steps: (1) predilution to approximately 11 IU/mL using buffer solution from the Coamatic FVIII kit; (2) dilution to approximately 1 IU/mL using FVIII-deficient plasma; (3) dilution to approximately 0.005 IU/mL using the Coamatic FVIII kit buffer solution. Blank samples were prepared by dilution of 20-µL FVIII-deficient plasma with 4,000-µL buffer solution.

The test samples (together with the calibrator and blank samples) were analyzed in triplicate on the ACL Elite Pro analyzer. This resulted in a total of six results for each time point. Absorbance readings for test samples and calibrator were used to calculate potency using a slope–ratio analysis. Potency at each time point was deemed acceptable if the mean potency (of all six results) for the test samples did not deviate by more than 20% of the mean potency of the reference sample (sample taken at 0 hours), in accordance with data obtained in similar studies. 28

### Purity

The purity of turoctocog alfa samples was assessed using reverse-phase high performance liquid chromatography (RP-HPLC). Analysis was performed on an HPLC system equipped with processing software and a 4.0 mm × 250 mm, C4, 5 μm, and 300-Å column (Novo Nordisk Pharmatech A/S, Køge, Denmark). The column temperature was set at 40°C, with a detection wavelength of 215 nm. A gradient of 35 to 100% eluent B (0.09% trifluoracetic acid [TFA] in 80% acetonitrile in purified water) and 65 to 0% eluent A (0.1% TFA in purified water) was applied over a 40-minute period with a flow rate of 1 mL/min. The composition of 100% eluent B was then maintained for 5 minutes, before being changed back to the initial conditions over 1 minute, followed by column equilibration for 14 minutes, resulting in a total run time of 60 minutes.

The purity of turoctocog alfa was calculated as the sum of area percentages of the following components on the resulting chromatograms: turoctocog alfa light chain, turoctocog alfa single chain, and three heavy-chain (HC) components (nontruncated form, one with the C-terminal at amino acid 740 [HC_740], and one with the C-terminal at amino acid 720 [HC_720]).

### Oxidized Forms

Oxidized forms within the turoctocog alfa samples were assessed using the same RP-HPLC system, column, HPLC parameters (column temperature, detection wavelength, and mobile phase eluents), and elution gradients as those used to assess product purity. Oxidized forms were calculated as the percentage area on the resulting chromatograms.

### Total High Molecular Weight Proteins and Content of Turoctocog Alfa

Samples were analyzed by size exclusion-HPLC (SE-HPLC) to determine the presence of protein aggregates and of turoctocog alfa in the samples. SE-HPLC measurements were performed using an HPLC system equipped with a BioSep SEC S3000 7.8 mm × 300 mm, 5 μm, 290-Å column (Phenomenex, Torrance, California, United States), or a Shodex PROTEIN KW-803. 8 mm × 300 mm column (Shodex, Showa Denko, Japan), or equivalent. An elution flow rate of 0.4 mL/min was employed, using a column temperature of 30°C and excitation and emission detection wavelengths of 285 and 335 nm, respectively. The eluent buffer consisted of 10 mM TRIS, 10 mM CaCl _2_ , 300 mM NaCl, and 5% 2-propanol at pH 7.0, and the injection volume was 100 µL, independent of protein concentration. Run times were ≥70 minutes. Total HMWP content was determined by calculating the area percentage of the HMWP peak on the resulting chromatogram. The content of turoctocog alfa was calculated from the area of the monomer peak, relative to the average monomer peak area from the five injections of the reference material (a one-point calibration).

## Results

### Appearance/Clarity of Solution and pH

There were no changes in clarity, color, or the appearance of foreign insoluble matter compared with the visual description at study start for any vial strengths or pump speeds evaluated. All samples complied with the acceptance criteria on visual inspection: each reconstituted sample appeared as a clear or slightly opalescent solution. At 24 hours, pH had not changed and remained at 6.8 for all samples.

### Potency

**Fig. 1:**
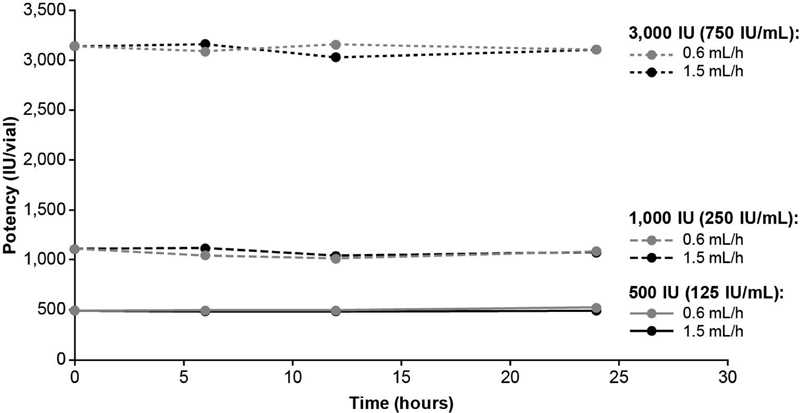
Potency of turoctocog alfa assessed during 24 hours of CI at pump speeds of 0.6 and 1.5 mL/h. The following turoctocog alfa samples were assessed: 500 IU (equating to 125 IU/mL), 1,000 IU (equating to 250 IU/mL), and 3,000 IU (equating to 750 IU/mL). The potency results for the test samples were all within the predefined acceptance criteria (within 20% of the mean potency of the sample of *t* = 0 hours). For the 1.5 mL/hour pump speed, the % difference from the mean potency at *t* = 0 hours was −1.57, −1.58, and –0.16 for 500-IU samples; 0.71, −5.96, and −2.87 for 1,000-IU samples; and 0.69, −3.50, and −1.12 for 3,000-IU samples. For the 0.6 mL/hour pump speed, the % difference from the mean potency at *t* = 0 hours was 0.42, 0.43, and 5.99 for 500-IU samples; −5.72, −8.49, and −2.15 for 1,000-IU samples; and −1.60, 0.63, and −1.11 for 3,000-IU samples. CI, continuous infusion.

At *t* = 0 hours, the mean potency (IU/vial) was: 491.6 for 500 IU, 1,108.0 for 1,000 IU, and 3,139.5 for 3,000 IU. The potency of turoctocog alfa was retained during 24 hours of CI for all vial strengths and both infusion speeds evaluated ( Fig. 1 ). Furthermore, the potency results for the test samples were all within the predefined acceptance criteria (within 20% of the mean potency of the *t* = 0 hours sample).

### Purity

**Fig. 2:**
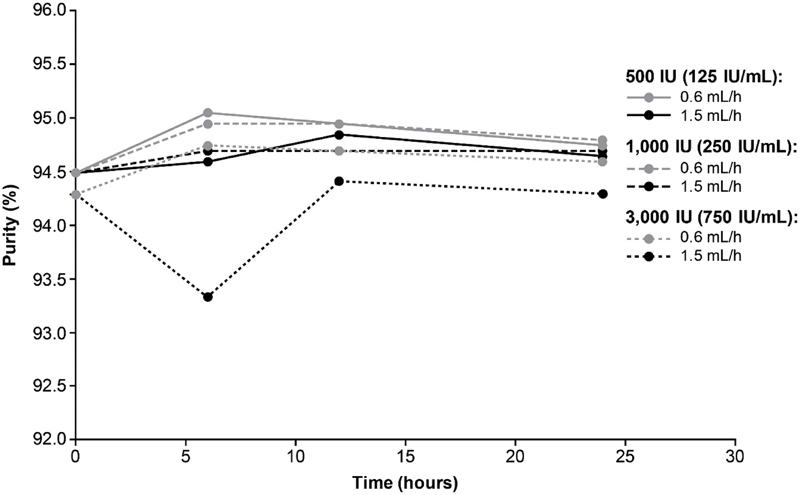
Purity of turoctocog alfa assessed during 24 hours of CI at pump speeds of 0.6 and 1.5 mL/h. The following turoctocog alfa samples were assessed: 500 IU (equating to 125 IU/mL), 1,000 IU (equating to 250 IU/mL), and 3,000 IU (equating to 750 IU/mL). CI, continuous infusion.

The purity of turoctocog alfa at *t* = 0 hours was 94.4 and 94.5% for 500 IU, 94.4 and 94.5% for 1,000 IU, and 94.3% (reading obtained for both replicates) for 3,000 IU. For all strengths of turoctocog alfa and both pump speeds evaluated, the mean purity was comparable to the reference samples ( *t* = 0) at 6, 12, and 24 hours of CI ( Fig. 2 ) and within predefined acceptance limits.

### Content of Turoctocog Alfa

**Fig. 3:**
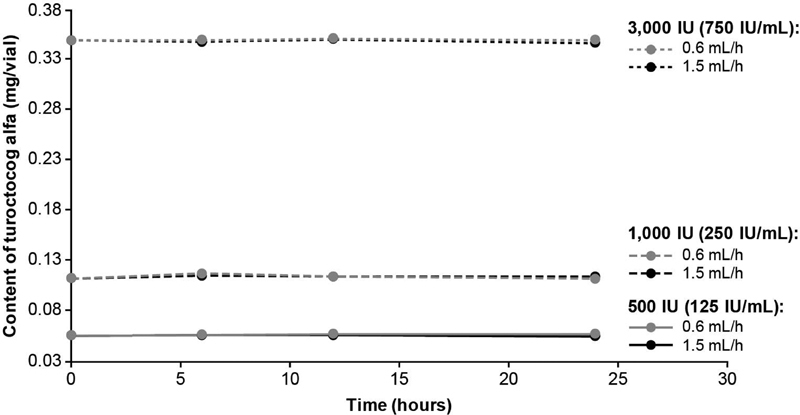
Turoctocog alfa content was assessed during 24 hours of CI at pump speeds of 0.6 and 1.5 mL/h. The following turoctocog alfa samples were assessed: 500 IU (equating to 125 IU/mL), 1,000 IU (equating to 250 IU/mL), and 3,000 IU (equating to 750 IU/mL). CI, continuous infusion.

At *t* = 0 hours, the turoctocog alfa content (mg/vial) was 0.055 and 0.056 for 500 IU, 0.112 and 0.113 for 1,000 IU, and 0.348 and 0.350 for 3,000 IU. The content of turoctocog alfa in the test samples was similar over 24 hours of CI for all vial strengths and both infusion speeds evaluated and within acceptance limits for shelf life ( Fig. 3 ).

### Total High Molecular Weight Proteins

**Fig. 4:**
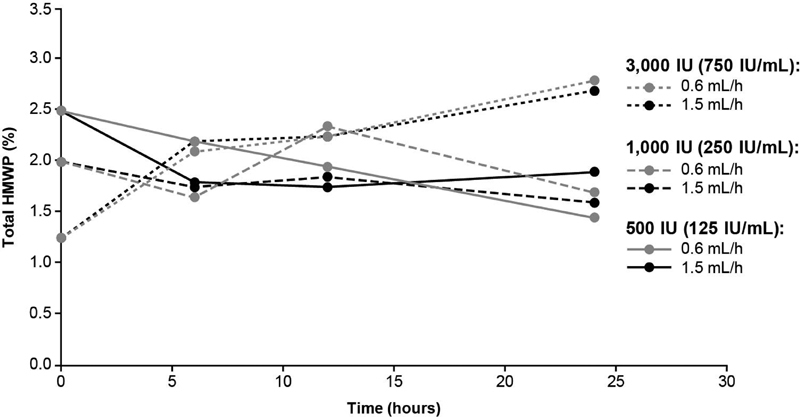
The total HMWP content of turoctocog alfa was assessed during 24 hours of CI at pump speeds of 0.6 and 1.5 mL/h. The following turoctocog alfa samples were assessed: 500 IU (equating to 125 IU/mL), 1,000 IU (equating to 250 IU/mL), and 3,000 IU (equating to 750 IU/mL). CI, continuous infusion; HMWP, high molecular-weight protein.

There were minor increases in total HMWP content over time, with the greatest increase in the 3,000-IU sample ( Fig. 4 ). The mean (%) for the 3,000-IU sample at 0, 6, 12, and 24 hours infusion was 1.3, 2.2, 2.3, and 2.7%, for the 1.5 mL/h pump speed, and 1.3, 2.1, 2.3, and 2.8% for the 0.6 mL/h pump speed, respectively. For all turoctocog alfa strengths tested, the mean total HMWP content at 6, 12, and 24 hours, for both pump speeds assessed, were comparable with the samples of *t* = 0 hours (2.5% for 500 IU, 2.0% for 1,000 IU, and 1.3% for 3,000 IU) and within acceptance criteria for shelf life.

### Oxidized Forms of rFVIII

**Fig. 5:**
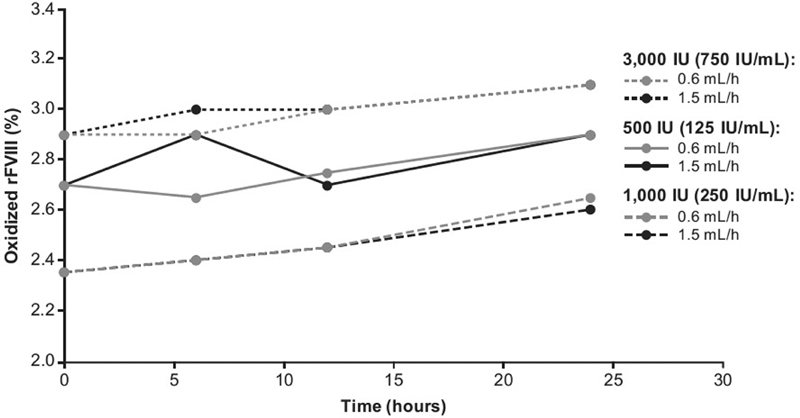
Oxidized forms of turoctocog alfa were assessed during 24 hours of CI at pump speeds of 0.6 and 1.5 mL/h. The following turoctocog alfa samples were assessed: 500 IU (equating to 125 IU/mL), 1,000 IU (equating to 250 IU/mL), and 3,000 IU (equating to 750 IU/mL). CI, continuous infusion; rFVIII, recombinant factor VIII.

There were small increases in oxidized forms of rFVIII over time ( Fig. 5 ), but for both pump speeds evaluated, the mean values at 6, 12, and 24 hours were comparable with those from the *t* = 0 hour samples (2.7% for 500 IU, 2.4% for 1,000 IU, and 2.9% for 3,000 IU) and within acceptance criteria.

## Discussion

A key requirement of FVIII concentrates for CI is that they are stable under the conditions of administration and over the duration of administration. Stability studies have demonstrated the enhanced stability of recombinant factor products following reconstitution at room temperature, from 24 hours to 7 days. 10 21 Continuous infusion has also been shown to be safe in the hospital setting and to provide a constant level of FVIII by balancing input with clearance. 4 6 7 8 9 14 Continuous infusion, therefore, provides an attractive alternative to bolus infusion, especially if replacement therapy is required for more than 3 days, for example, during surgery or severe bleeds which require hospitalization. 14 Other than cost savings, 13 the benefits of continuous infusion avoids peak FVIII levels which can occur in patients with comorbidities, such as age, obesity, thrombotic risk, and trough FVIII levels, associated with high bleeding risk. 29 30

Here, we have shown that the chemical and physical stability of turoctocog alfa was maintained during CI at 30°C over 24 hours, under the conditions assessed. There was no degeneration or change in the potency, purity, or content of turoctocog alfa, and only minor increases in total HMWP and oxidized forms, across a range of turoctocog alfa strengths (500, 1,000, and 3,000 IU) equating to doses of 1.1 to 16.1 IU/h/kg of body weight. In all cases, results were similar to those of the sample of *t* = 0 hours and, for potency, within the prespecified acceptance criteria.

Continuous infusion administration practices vary across centers and regions. 13 14 In this study, we used three concentrations of turoctocog alfa, at a low- and high-pump speeds, using methods for CI aligned with those used in clinical practice centers in Japan and the United Kingdom. It has been recommended that replacement products should not be further diluted for CI to avoid affecting product stability and to allow a low volume for infusion. 14 However, many centers do dilute factor concentrates and report stable FVIII activity levels. 28 31 In the interest of safety, it is recommended to test the stability of factor concentrates with the particular infusion set for CI. 14

## Limitations

As this is an in vitro study, one limitation is that it cannot replicate all situations that may affect FVIII concentrate stability in the clinical setting. For example, CI relies on good venous access; any failure in venous access in practice may lead to a reduction in infusion time and a risk of reduced FVIII level. Furthermore, no precautions were taken during the in vitro analysis to protect the reconstituted turoctocog alfa solution from direct light (as advised by the manufacturer). 17

## Conclusion

The stability of turoctocog alfa was maintained during CI at 30°C over 24 hours, with no degradation or change in any of the chemical and physical parameters tested as per predefined acceptance criteria consistent with shelf life specifications. Particularly, the potency of turoctocog alfa was sustained throughout the 24 hours of CI.
